# Manganese-porphyrin-enhanced MRI for the detection of cancer cells: A quantitative *in vitro* investigation with multiple clinical subtypes of breast cancer

**DOI:** 10.1371/journal.pone.0196998

**Published:** 2018-05-24

**Authors:** Mosa Alhamami, Weiran Cheng, Yuanyuan Lyu, Christine Allen, Xiao-an Zhang, Hai-Ling Margaret Cheng

**Affiliations:** 1 Department of Pharmaceutical Sciences, Leslie Dan Faculty of Pharmacy, University of Toronto, Toronto, Ontario, Canada; 2 Institute of Biomaterials and Biomedical Engineering, University of Toronto, Toronto, Ontario, Canada; 3 Translational Medicine, Hospital for Sick Children Research Institute, Toronto, Ontario, Canada; 4 Department of Chemistry, University of Toronto, Toronto, Ontario, Canada; 5 Department of Physical and Environmental Sciences, University of Toronto Scarborough, Toronto, Ontario, Canada; 6 School of Medicine, Zhejiang University City College, Hangzhou, Zhejiang, P. R. China; 7 Spatio-Temporal Targeting and Amplification of Radiation Response (STTARR) Innovation Centre, Radiation Medicine Program, Princess Margaret Cancer Centre, University Health Network, Toronto, Ontario, Canada; 8 Department of Biological Sciences, University of Toronto Scarborough, Toronto, Ontario, Canada; 9 The Edward S. Rogers Sr. Department of Electrical and Computer Engineering, University of Toronto, Toronto, Ontario, Canada; 10 Translational Biology and Engineering Program, Ted Rogers Centre for Heart Research, Toronto, Ontario, Canada; Brandeis University, UNITED STATES

## Abstract

Magnetic resonance imaging (MRI) contrast agents (CAs) are chemical compounds that can enhance image contrast on T_1_- or T_2_- weighted MR image. We have previously demonstrated the potential of MnCl_2_, a manganese-based CA, in cellular imaging of breast cancer using T_1_-weighted MRI. In this work, we examined the potential of another class of manganese-based CAs, manganese porphyrins (MnPs), for sensitive cellular detection of multiple clinical subtypes of breast cancer using quantitative MRI. Using a clinical 3.0-T MRI scanner, the relaxivities of two MnPs, MnTPPS_4_ and MnTPPS_3_NH_2_, and conventional Gd-DTPA (control) were measured in ultrapure water and their T_1_ contrast enhancement patterns were characterized in multiple clinical subtypes of breast cancer. The toxicity of the three CAs was evaluated *in vitro*. Compared to Gd-DTPA, both MnTPPS_3_NH_2_ and MnTPPS_4_ enabled a more sensitive multi-subtype detection of four breast cell lines at doses that posed no cytotoxic effects, with MnTPPS_3_NH_2_ producing the greatest positive enhancement. The superior T_1_ enhancement capabilities of MnPs over Gd-DTPA are statistically significant and are likely due to their greater cellular uptake and relaxivities. The results demonstrate that multiple clinical subtypes of breast cancer can be imaged on a 3.0-T MRI scanner using MnPs as T_1_ cellular CAs.

## Introduction

Clinically, gadolinium–diethylenetriamine pentaacetic acid (Gd-DTPA)-enhanced MRI is used, in conjunction with X-ray mammography, for screening women who are at high risk (lifetime risk ≥20–25%) of developing breast cancer [1]. Gd-DTPA-enhanced MRI is also relied upon routinely to delineate vasculature and tumor extent once suspicious findings are initially identified with X-ray mammography. However, as a T_1_ contrast agent (CA), Gd-DTPA is associated with a number of critical limitations. These include toxicity due to potential dissociation of Gd from the DTPA chelator [2], relativity low relaxivity [3], and rapid distribution into and wash out from malignant sites. In fact, due to the low tumor retention and temporally diminished contrast effect of Gd-DTPA, its post-contrast imaging window is often limited to few minutes [1,4].

Engineering CAs that can be taken up and retained by cells could enable sensitive detection and visualization of labelled cells over a relatively long (e.g. ≥ 20 hrs) imaging window, thereby providing information on physiological processes at the cellular level. Cellular CAs can either shorten the longitudinal relaxation time T_1_ (positive enhancement) or the transverse relaxation time T_2_ (negative enhancement). T_1_-shortening cellular CAs increase signal intensity (i.e. produce image brightening) with T_1_-weighted MRI parameters, while T_2_-shortening cellular CAs decrease signal intensity (i.e. produce image darkening) with T_2_- or T_2_*-weighted MRI parameters [5]. However, a fundamental drawback of T_2_-shortening CAs in MRI is the lack of specificity; distinction of T_2_ CA-labelled cells is difficult due to the intrinsic low signal intensity or negative contrast that may arise from some background, CA-free biologic tissues. Therefore, it is highly desirable to design and evaluate T_1_ CAs that can be efficiently internalized by cells. By virtue of their cellular uptake and retention within the tumor site, these CAs can provide sensitive and persistent identification of labelled cells, improving tumor imaging window even in less vascularized masses. Additionally, cellular CAs, unlike Gd-DTPA, can allow sensitive detection of cancer cells directly at an early stage, when vasculature has not been fully formed at tumor site. In fact, recent work using MnCl_2_ as a T_1_ cellular CA demonstrated the potential of MRI as a cellular imaging modality that is capable of directly detecting breast cancer cells *in vitro* [6] as well as characterizing the metastatic potential of early small (≤5 mm^3^) breast tumors in mice [7]. Moreover, the same research group demonstrated that larger, more advanced breast tumors that did not enhance on Gd-DTPA underwent substantial enhancement with MnCl_2_ [8].

Manganese porphyrin (MnP) is another class of Mn-based CAs that can produce positive enhancement on T_1_-weighted MRI [9–11] with multiple routes of administration, including intravenous injections [12]. MnPs offer several advantages over conventional Gd-DTPA. First, because the porphyrin ring binds Mn^3+^ with high stability, the likelihood of ion release in MnPs is very low. In fact, the most extensively investigated MnP, manganese (III) tetraphenyl porphyrin sulfonate (MnTPPS_4_), exhibited no demetallation *in vitro* in human plasma for up to 9 days [13] and only about 1% degree of demetallation *in vivo* in liver and kidney up to 4 days post administration [14]. Additionally, MnPs have higher relaxivities than Gd-DTPA [12,15]; specifically, MnTPPS_4_ has recently been shown to have a lower minimum detectable concentration and a greater sensitivity than Gd-DTPA in phosphate buffered saline [16]. Therefore, MnPs can achieve a positive contrast enhancement similar to Gd-DTPA but at lower doses, which reduces their systemic toxicity. Previous studies have specifically explored the potential of MnTPPS_4_ as a tumor-seeking agent. In addition to tumor characteristics (high capillary permeability, poor lymphatic drainage, and large interstitial fluid space), Fiel *et al*. attributed the localization of MnTPPS_4_ in L1210 solid tumors grown in mice to the size of the planar porphyrin ring, favoring its retention and aggregation in tumors [14]. Also, Fiel *et al*. demonstrated cellular uptake of MnTPPS_4_ in L1210 tumors *in vivo* and noted that by increasing the hydrophobicity of porphyrins, they would be transported across plasma membranes more readily [14]. Shortly thereafter, Place *et al*. reported the greater uptake of MnTPPS_4_ in MCF-7 breast cancer cells (× 175 after 24 hrs) compared to a normal breast cell line (Hs578Bst) *in vitro* [17]. The temporally regulated tumor retention efficacy of MnTPPS_4_ was also demonstrated *in vivo* in several tumor models [12,18,19]. However, there has been no experimental evidence pertaining to the capability of MnTPPS_4_-enhanced MRI in detecting multiple clinical subtypes of breast cancer.

The purpose of this work is to determine whether MnTPPS_4_ is capable of directly detecting multiple clinical subtypes of breast cancer cells (Table 1) compared to Gd-DTPA, used previously in cell labelling studies [20–22]. This work also aims at determining whether an amino MnP, known as MnTPPS_3_NH_2_ (see S1 Fig), can provide more sensitive cellular detection of breast cancer cells than MnTPPS_4_, thereby lowering the dose needed to achieve positive enhancement on T_1_-weighted MR image.

**Table 1:** The clinical subtypes and molecular classification of breast cancer cell lines used in this study (adapted from references [24,25]).

Cell line	Classification / subtype	Tumorigenicity	Receptor expression
MDA-MB-231	Claudin low[24]; basal-like[25]	Tumorigenic	ER ^-^, PR ^-^, HER2 ^-^
MCF-7	Luminal A[24,25]	Tumorigenic	ER ^+^, PR ^+/-^, HER2 ^-^
ZR-75-1	Luminal B[24]; luminal A[25]	Tumorigenic	ER ^+^, PR ^+/-^, HER2 ^+/-^
MCF-10A	Basal-like[25]	Non-tumorigenic	ER ^-^, PR ^-^, HER2 ^-^

ER, estrogen receptor; PR, progesterone receptor; HER2, human epidermal growth factor receptor 2.

Zhang *et al*. previously synthesized MnTPPS_3_NH_2_ and conjugated it to dextran [23]. Compared to Gd-DTPA, MnTPPS_3_NH_2_-dextran conjugate enabled a greater and more prolonged signal enhancement on T_1_-weighted MRI, possibly due to the binding of dextran to cell membranes which facilitates the targeting of MnTPPS_3_NH_2_ to tumor cells [23]. However, to the best of our knowledge, no previous work has investigated the potential of the unconjugated MnTPPS_3_NH_2_ as a T_1_ CA for sensitive detection of multiple clinical subtypes of breast cancer cells using quantitative MRI.

It is hypothesized that MnP-enhanced MRI is capable of enabling a more substantial enhancement of various subtypes of breast cancer cells compared to conventional Gd-DTPA-enhanced MRI. It is further hypothesized that MnTPPS_3_NH_2_ is a more effective T_1_ CA than MnTPPS_4_ for multi-subtype detection of breast cancers using quantitative MRI due to its higher hydrophobicity and cellular uptake.

## Materials and methods

### Contrast agent solutions preparation and sterilization

To make the first stock solution of the CAs, autoclaved, ultrapure water (MilliQ Purification System, Millipore, Darmstadt, Germany) was added to MnTPPS_4_ (5,10,15,20-Tetrakis(4-sulfonatophenyl)-21H,23H-porphine manganese(III) chloride, Sigma-Aldrich Canada Co., Oakville ON, Product# 441813) and MnTPPS_3_NH_2_ (synthesized in our laboratory). The CA solutions were filtered using a 33-mm-diameter sterile Millex-GP syringe filter unit with a 0.22-μm pore size hydrophilic polyethersulfone membrane (SLGP033RS, Millipore, Darmstadt, Germany). The first stock solutions were sono-bathed in an ultrasonic cleaner (VWR symphony, VWR International, Mississauga, Canada) for 30 minutes at about 65 ^o^C. To further sterilize and solubilize the stock solutions, they were placed at a hotplate, preheated at 100–200 ^o^C, for approximately 5–7 minutes. Serial dilutions (1000X) were made inside a biosafety cabinet in order to decrease the concentrations of the sterilized first stock solutions of the CAs to detectable levels on a UV-visible spectrophotometer. The concentrations of the diluted solutions were then determined by measuring absorbance using a UV-visible spectrophotometer. Knowing the dilution factors, the concentrations of the first stock solutions of the CAs were found.

### Relaxivity measurements

Stock solutions of the three CAs used in this study, MnTPPS_4_, MnTPPS_3_NH_2_ and Gd-DTPA (Magnevist, Bayer HealthCare LLC, Whippany, NJ), were prepared in ultrapure MilliQ water at concentrations 0, 0.05, 0.1 and 0.2 mM per metal ion. The solutions were transferred to 115 × 5 mm wintrobe sedimentation tubes (Kimble Chase, Vinelad, NJ). A total of 30 sedimentation tubes were loaded with CA solutions at the aforementioned concentrations (three tubes for each CA dissolved at a given concentration). The sedimentation tubes were then placed in an upright position in an ultem resin (McMaster-Carr, Chicago, IL) holder and transferred to the MRI suite. The ultem resin holder, with the sedimentation tubes containing CA solutions, was placed within a 32-channel receive/transmit head coil. MRI was performed on a 3.0-T MRI scanner (Achieva 3.0 T TX, Philips Medical Systems, Best, the Netherlands). Longitudinal and transverse relaxivities, r_1_ and r_2_, were determined by measuring T_1_ and T_2_ relaxation times for each CA solution at the aforementioned concentrations and calculating the slope of the linear regression model fitted on a 1/T_1_ (longitudinal relaxation rate) or 1/T_2_ (transverse relaxation rate) versus CA concentration, [CA]. CA-induced changes in longitudinal and transverse relaxation rates are given by the following mathematical expressions, where $\frac{1}{T_{1}^{'}}$ and $\frac{1}{T_{2}^{'}}$ are intrinsic relaxation rates without CA:

$$\frac{1}{T_{1}}=\frac{1}{T_{1}^{'}}+r_{1}\times [CA]$$ (1)

$$\frac{1}{T_{2}}=\frac{1}{T_{2}^{'}}+r_{2}\times [CA]$$ (2)

To measure T_1_ relaxation times, a two-dimensional (2D) inversion-recovery turbo spin-echo (IR-TSE) sequence was utilized with the following parameters: inversion times (TI) = [50, 100, 250, 500, 750, 1000, 1250, 1500, 2000, 2500] ms, repetition time (TR) = 3000 ms, echo time (TE) = 18.5 ms, slice thickness = 7 mm, field-of-view (FOV) = 60 × 60 mm, in-plane resolution = 0.5 × 0.5 mm, and TSE factor = 4. T_2_ relaxation times were measured using a multi-spin-multi-echo (MSME) spin-echo sequence with the following parameters: TR = 2000 ms, 32 echoes with TE = [7.6, 15.3, 22.9 … 244.0], slice thickness = 7 mm, FOV = 60 × 60 mm, and in-plane resolution = 0.5 × 0.5 mm.

### Cell culture

The human breast cancer cell lines MDA-MB-231, MCF-7, and ZR-75-1 as well as a non-tumorigenic breast epithelial cell line, MCF-10A, were obtained from ATCC (American Tissue Culture Collection, Manassas, VA) and used in this study. The cells were maintained at 37 ^o^C with 5% CO_2_ in their recommended culture media: MDA-MB-231 in high glucose-containing Dulbecco’s Modification of Eagle’s Medium (DMEM), 1X (cat. # 319-005-CL, Wisent Inc., Saint-Jean-Baptiste, QC, Canada); MCF-7 in Eagle’s Minimum Essential Medium (EMEM) (ATCC cat. # 30–2003) supplemented with 0.01 mg/mL bovine insulin (Sigma-Aldrich cat. # I0516); ZR-75-1 in RPMI-1640 (ATCC cat. # 30–2001); MCF-10A in Mammary Epithelial Base Medium (MEBM) supplemented with additives (Lonza MEGM kit cat. # CC-3150). These additives, which are provided with the kit, include 2 mL bovine pituitary extract (BPE), 0.5 mL human epidermal growth factor (hEGF), 0.5 mL hydrocortisone, and 0.5 mL insulin. In addition, 100 ng/mL cholera toxin (Sigma-Aldrich cat. # C8052) was added to make the complete growth medium for MCF-10A, as per ATCC recommendations. The aforementioned four growth media were supplemented with 10% (v/v) fetal bovine serum (FBS) (ATCC cat. # 30–2020).

The cultures were initiated by thawing an ATCC cryo-vial that had been stored in liquid nitrogen (vapor phase) and plating the cells in a T-25 flask (Sarstedt Inc., Montreal, QC, Canada). When the flask was 80–90% confluent, the cells were passaged to T-175 flasks after washing them once with Dulbecco’s Phosphate Buffered Saline (DPBS, without calcium and magnesium), 1X (ATCC cat. # 30–2200) and adding trypsin-EDTA solution, 1X (0.25% trypsin / 0.53 mM ethylenediaminetetraacetic acid) without calcium and magnesium (ATCC cat. # 30–2101) for 4–5 minutes (MDA-MB-231, MCF-7, and ZR-75-1) or 0.05% trypsin / 0.02% EDTA (ATCC cat. # PCS-999-003) for at least 20 minutes (MCF-10A). The trypsin-EDTA solution was deactivated by adding a serum-supplemented medium. Prior to plating in new flasks, the cells were centrifuged at 125 *xg* at room temperature for 5 minutes. The supernatant containing the deactivated trypsin-EDTA and medium mixture was aspirated and replaced with fresh, serum-supplemented medium.

### Cell labelling with CAs and preparation for MRI

#### Labelling MDA-MB-231 cells at varying CA concentrations: CA direct exposure and retention experiments

The initial fundamental experiments evaluated the three CAs with an aggressive, triple-negative breast cancer cell line, MDA-MB-231, at varying concentrations and incubation conditions (CA direct exposure and cellular retention). Solutions of the CAs were added to MDA-MB-231 complete growth medium (DMEM + 10% FBS) at 0.05, 0.1 and 0.2 mM. The CA-containing complete growth media were then shaken to ensure uniform distribution and were administered to 9 T-175 flasks (3 for each CA, covering the three concentrations) of MDA-MB-231 cells growing in the exceptional growth phase. The 9 CA-containing T-175 flasks and another CA-free T-175 flask (control), which had also undergone a medium change at the time of CA administration, were incubated for up to 23 hours at 37 ^o^C in 5% CO_2_. Subsequently, the cells were rinsed once with DPBS, trypsinzed with trypsin-EDTA solution for about 4 minutes, and centrifuged at 125 *xg* at room temperature for 5 minutes. Because the trypsin-EDTA solution was deactivated with the original CA-containing medium in each one of the 9 T-175 flasks, the cells were rinsed again with DPBS immediately following the aspiration of the supernatant. This step was followed by another centrifugation, also at 125*g* at room temperature for 5 minutes, the removal of the DPBS supernatant, and the addition of a fresh, serum-free growth medium (i.e. only DMEM) to each centrifuge tube. The cell suspensions from the 10 T-175 flasks were collected in ten separate 50-mL conical centrifuge tubes and were subsequently transferred to 115 × 5 mm Wintrobe sedimentation tubes. Each one of the 10 sedimentation tubes was placed in a larger 15-mL centrifuge tube and spun, for the last time prior to MRI, at 440 *xg* at room temperature for 15 minutes to create cell pellets for MRI. The opening of each sedimentation tube was secured firmly with a tape.

The aforementioned procedures were performed on two sets of 10 T-175 flasks: the CA direct-exposure set and the CA cellular retention set. In the CA direct-exposure set, the cells were incubated with the CAs for up to 23 hours at 37 ^o^C in 5% CO_2_ and cell pellets were prepared for MRI immediately following the removal of the CA-containing media as described above. In the CA cellular retention set, after 23–24 hours of incubation with the CAs at 37 ^o^C in 5% CO_2_, the cells were rinsed twice with DPBS following the removal of the CA-containing media from the T-175 flasks, supplemented with fresh, CA-free complete growth medium (DMEM + 10% FBS), and allowed to grow in CA-free medium for an additional 24–27 hours at 37 ^o^C in 5% CO_2_ to examine the differential capability of the three CAs in being retained intracellularly at varying incubation concentrations. At the end of the incubation period, cell pellets for the CA cellular retention set were prepared for MRI as previously described.

#### CA labelling of multiple clinical subtypes of breast cancer

In order to evaluate the capability of the proposed quantitative cellular MRI approach for sensitive detection of multiple clinical subtypes of breast cancer, the four breast cell lines (Table 1) were labelled with the three CAs at 0.2 mM following the aforementioned CA direct-exposure labelling protocol at approximately a 24-hr labelling interval. A control (unlabelled) cell pellet was also prepared for each cell line.

### Cytotoxicity assessment of CA-labelled breast cell lines

Cell viability of CA-labelled breast cell lines was assessed by trypan blue exclusion method with an automated cell viability analyzer (Vi-CELL XR cell viability analyzer, Beckman Coulter Inc., Mississauga, Canada) to determine whether labelling with any one of the three CAs had cytotoxic effects on the four breast cell lines. Briefly, cells were plated in 96-well assay plates (Corning Costar, Corning, NY) at a cell density of about 15–20 × 10^3^ cells/well, thoroughly suspended in a medium volume of 150 μL, using an X-stream repeater pipette (Eppendorf, Mississauga, Canada) such that there are five replicates for the cells that would later be labelled with Gd-DTPA, MnTPPS_4_, or MnTPPS_3_NH_2_. Another two 5-well replicates were plated around the 15 CA wells for the unlabelled (control) cells (see S2 Fig). After seeding a total of 25 wells in each 96-well plate, the cells were incubated at 37 ^o^C in 5% CO_2_ to allow recovery from trypsinization and adherence to the culture plates. To avoid evaporation and edge effects, the 25 experimental wells in each 96-well plate for each cell line were placed in the middle of the plate, whereas all of the other remaining no-cell wells were filled with complete growth media at a volume slightly higher than the experimental wells (170 μL). After the two-day incubation period, cells were labelled with CAs at 0.2 mM for 24–27 hours at 37 ^o^C in 5% CO_2_. At the end of the labelling interval, cells from each well were washed with DPBS, trypsinized and harvested, as previously described, in 2-mL microtubes. The viability was then assessed using Vi-CELL viability analyzer for each well. Cell morphology was observed and mean diameter was measured using the analyzer.

### MRI of CA-labelled breast cancer cell lines

After preparing CA-labelled cell pellets, the sedimentation tubes were placed in an upright position in an ultem resin holder and transferred in a styrofoam ice box to the MRI suite. The ultem resin holder, with the sedimentation tubes containing cell pellets, was placed within a 32-channel receive/transmit head coil and MRI was performed on the 3.0-T MRI scanner.

Quantitative T_1_ and T_2_ relaxation times were measured using the same IR-TSE and MSME sequences utilized previously in the relaxivity measurements. However, due to the shallow depth of some cell pellets, the slice thickness had to be adjusted to 2.5 mm and 2.0 mm during the scans. High-resolution T_1_-weighted images were also acquired using a 2D spin-echo sequence with the following parameters: TR = 167 ms, TE = 14.2 ms, FOV = 60 × 60 mm, flip angle = 90^o^, number of signal averages = 8, in-plane resolution = 0.5 × 0.5 mm, and slice thickness = 2.5 mm (CA direct-exposure scan with MDA-MB-231 and multi-cell line scan) and 2.0 mm (CA cellular retention scan with MDA-MB-231).

### Quantitative MRI data analysis

Quantitative analysis of the MRI data was performed using in-house software developed in MATLAB (R2014a, version 8.3, MathWorks, Natick, MA). To calculate T_1_ and T_2_ relaxation times, regions of interest (ROIs) were outlined within each cell pellet- or CA solution-containing sedimentation tube in the MR images. Calculation of the relaxation times was performed on a pixel-by-pixel basis in each pellet/tube. To compute a quantitative map of T_1_ relaxation times, T_1_ was calculated at each pixel within an ROI by fitting the signal intensity versus TI at that pixel to the following function:

$$A\times \left|1-2\exp\left(-\frac{TI}{T_{1}}\right)+\exp\left(-\frac{TR}{T_{1}}\right)\right|$$ (3)

where A and T_1_ are free parameters.

A quantitative map of T_2_ relaxation times was computed by calculating T_2_ at each pixel within an ROI through fitting the signal intensity versus TE at that pixel to a mono-exponential decay function added to a constant offset to account for noise. The T_1_ and T_2_ relaxation times for each ROI within the breast cancer cell pellets represent mean values ± standard deviations from all the pixels within that ROI in the quantitative maps of T_1_ and T_2_, respectively. The longitudinal and transverse relaxivities, r_1_ and r_2_, were determined by linear regression analysis on the change in the inverse of relaxation times (1/T_1_ and 1/T_2_) versus CA concentration.

### Statistical analysis

To investigate the significance in the differences of means of relaxation times, a Student’s t-test was performed at the 95% confidence level (P = 0.05). The number of pixels in each cell pellet’s ROI was used as the sample size (n) in calculating the 95% confidence intervals for two-sample comparisons of relaxation times using in-house software developed in MATLAB.

## Results

### Longitudinal and transverse relaxivity measurements

**Fig 1:**
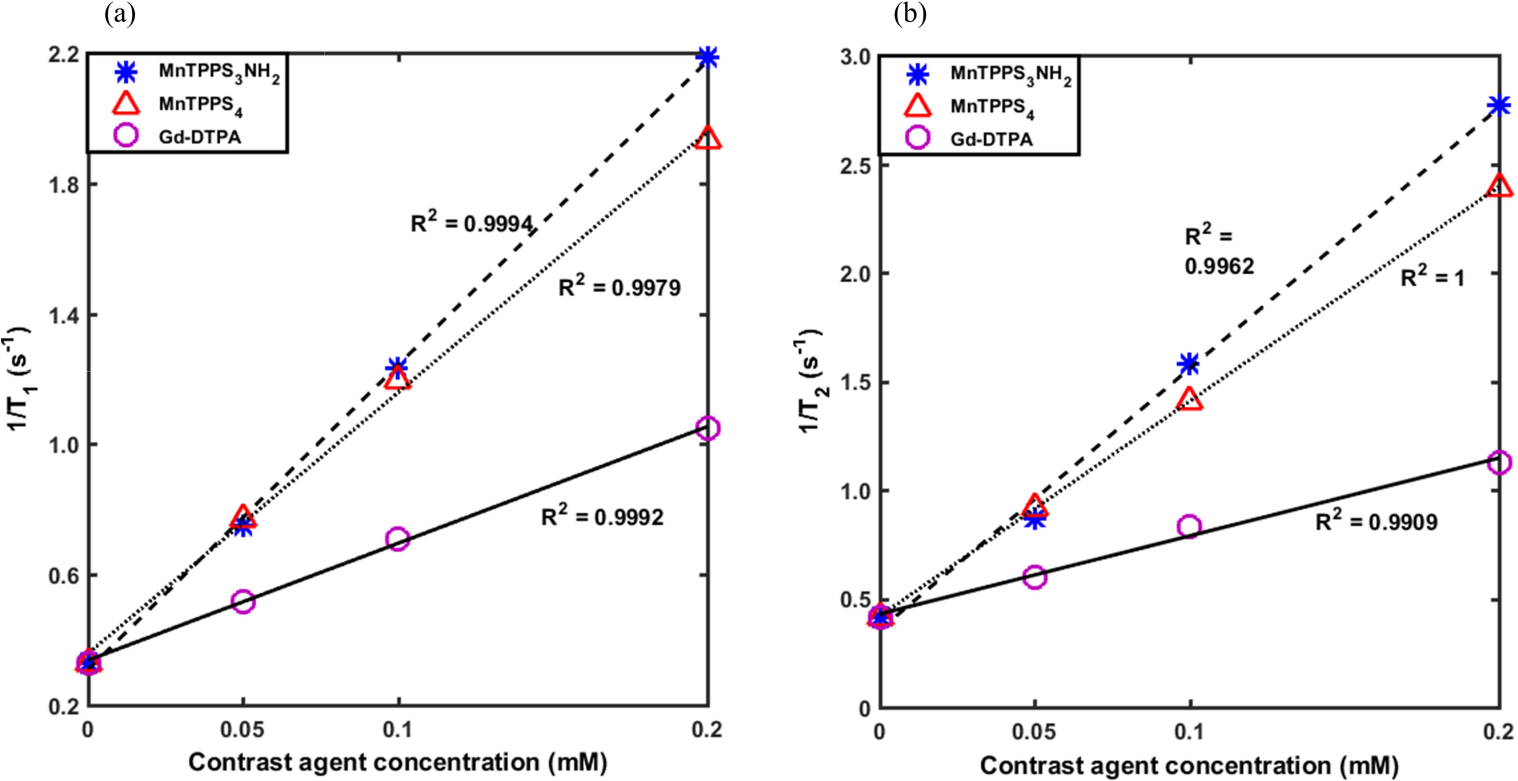
Relaxivity measurements. The measured (a) 1/T_1_ and (b) 1/T_2_ values as a function of CA concentration for the three CAs in MilliQ water at 3.0 T and room temperature. Shown also are the fitted linear regression lines and the corresponding R^2^ values.

Fig 1A and 1B show the 1/T_1_ (longitudinal relaxation rate) and 1/T_2_ (transverse relaxation rate) as a function of CA concentration for MnTPPS_3_NH_2_, MnTPPS_4_ and Gd-DTPA in MilliQ water at 3.0 T and room temperature. Shown also are the fitted linear regression lines along with the corresponding R^2^. Longitudinal and transverse relaxivity constants, r_1_ and r_2_, of the three CAs were calculated from the linear regression slopes of graphs (a) and (b), respectively, and are summarized in Table 2. A more than 2.5-fold and 2-fold increase in longitudinal relaxivity was attained for MnTPPS_3_NH_2_ (r_1_ = 9.33 mM^-1^ s^-1^) and MnTPPS_4_ (r_1_ = 7.99 mM^-1^ s^-1^), respectively, over Gd-DTPA (r_1_ = 3.58 mM^-1^ s^-1^). However, the r_2_/r_1_ ratios for the three CAs are close to unity at relatively low CA concentrations.

**Table 2:** Longitudinal and transverse relaxivity constants (r_1_ and r_2_) measured at 3.0 T at room temperature.

Contrast agent	r_1_	r_2_
MnTPPS_3_NH_2_	9.33	12.00
MnTPPS_4_	7.99	9.87
Gd-DTPA	3.58	3.58

Units for r_1_ and r_2_ are in mM^-1^ s^-1^.

### Quantitative MRI of MDA-MB-231 cells labelled with varying CA concentrations

#### CA direct exposure results

**Fig 2:**
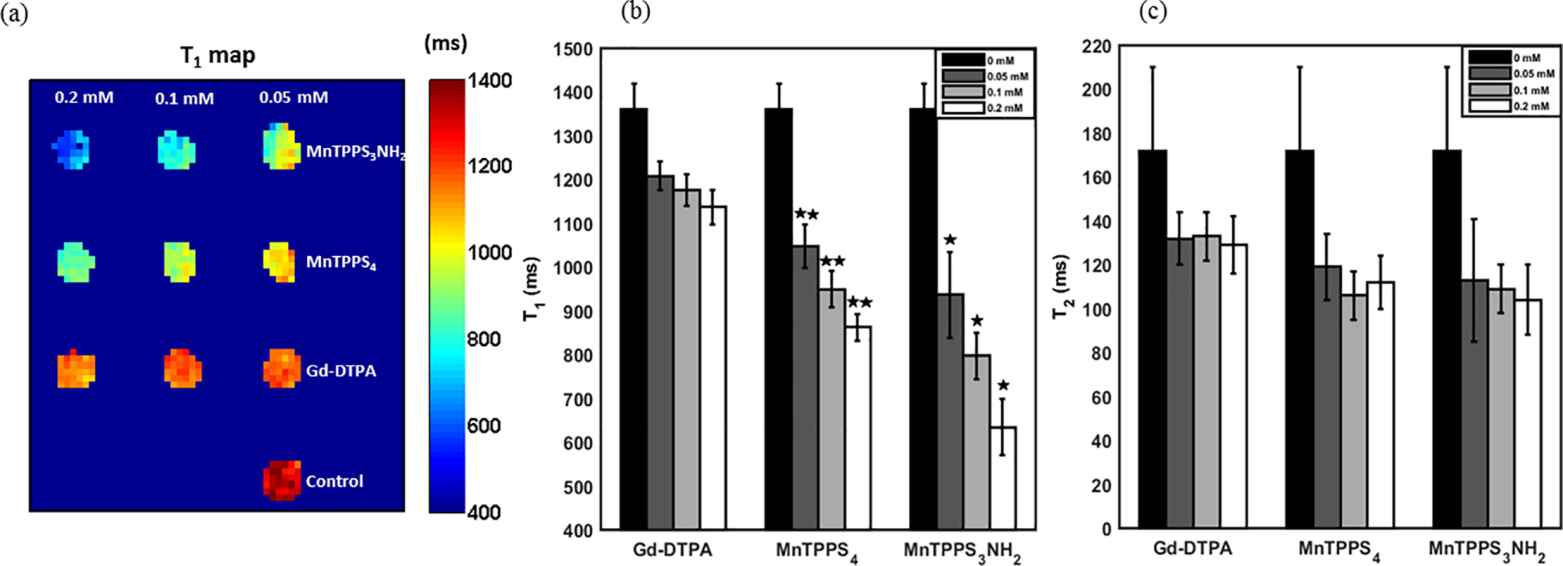
Quantitative MRI of CA direct exposure uptake in MDA-MB-231 cells. (a) A quantitative map of T_1_ relaxation times of the CA direct exposure cell pellets. (b) T_1_ and (c) T_2_ relaxation times of the CA direct exposure cell pellets. Shown are mean values ± standard deviation from all the pixels within each ROI in Fig 2A. *Indicates significant difference (P < 0.05) from MnTPPS_4_.** Indicates significant difference (P < 0.05) from Gd-DTPA.

Fig 2A shows a quantitative map of T_1_ relaxation times of MDA-MB-231 cell pellets following direct exposure to CAs. The largest decrease in T_1_ of the MnTPPS_3_NH_2_-labelled cell pellet at 0.2 mM (53.3%) corresponds to the greatest positive signal enhancement on T_1_-weighted MRI compared to the unlabelled cell pellet. MnTPPS_4_ provided a 36.6% reduction in T_1_, whereas Gd-DTPA offered only a 16.5% decrease in T_1_ at 0.2 mM, both relative to unlabelled cell pellet. The aforementioned differences are statistically significant at 95% confidence level.

Fig 2B and 2C show the averaged T_1_ and T_2_ relaxation times of the aforementioned CA labelled cell pellets. The values represent mean ± standard deviation from all the pixels within each ROI in the quantitative maps of T_1_, Fig 2A, and T_2_ relaxation times. For each CA, the difference among the T_1_ times at 0, 0.05, 0.1, and 0.2 mM is statistically significant (P < 0.05). At each CA concentration, the difference among the CAs is also statistically significant (P < 0.05). In fact, between 0.05–0.2 mM, MnTPPS_3_NH_2_ produced a 10.6% to 26.4% reduction in T_1_ compared to MnTPPS_4_, which enabled a further 13.2% to 24.1% reduction in T_1_ over Gd-DTPA. The greatest reduction in T_1_ times among the CAs is measured at 0.2 mM. On the other hand, the induced CA dose-dependent changes on T_2_ times of MDA-MB-231 cells are primarily insignificant at 95% confidence level for the three CAs.

#### CA cellular retention results

**Fig 3:**
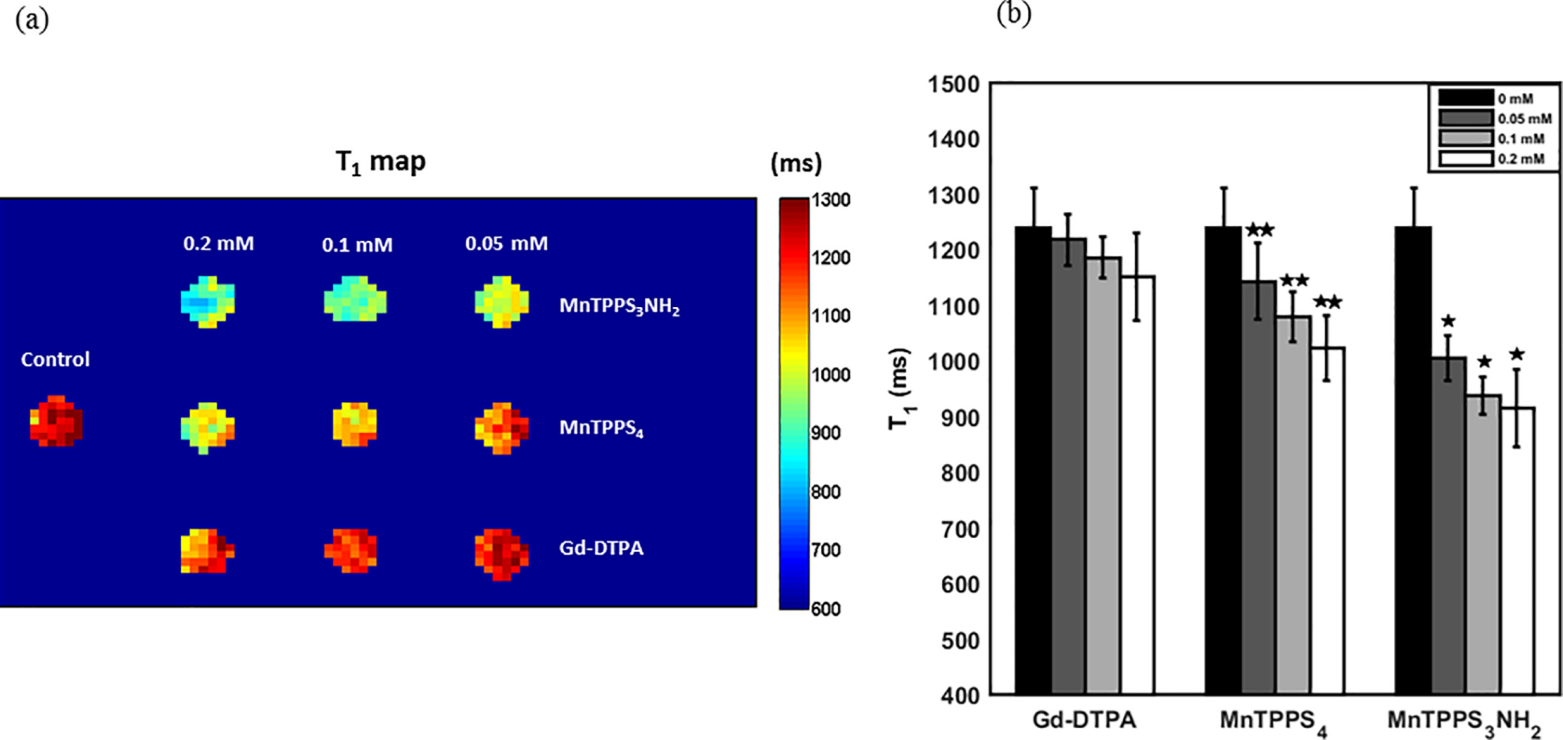
Quantitative MRI of CA retention in MDA-MB-231 cells. (a) A quantitative map of T_1_ relaxation times of CA cellular retention pellets imaged 24–27 hrs post-cell labelling. (b) T_1_ relaxation times of CA cellular retention pellets. Shown are mean values ± standard deviation from all the pixels within each ROI in Fig 3A. *Indicates significant difference (P < 0.05) from MnTPPS_4_.** Indicates significant difference (P < 0.05) from Gd-DTPA.

A quantitative map of T_1_ relaxation times of the CA cellular retention cell pellets is shown in Fig 3A. Fig 3B shows the averaged T_1_ relaxation times for the aforementioned cell pellets. The values represent mean ± standard deviation from all the pixels within each ROI in the quantitative map of T_1_ relaxation times, Fig 3A. The largest decrease in the averaged T_1_ time of the MnTPPS_3_NH_2_-labelled cell pellet at 0.2 mM (26.2%) corresponds to the greatest positive signal enhancement on T_1_-weighted MRI compared to the unlabelled cell pellet. MnTPPS_4_ provided a 17.4% reduction in T_1_ (P < 0.05), whereas Gd-DTPA enabled only a 7.1% decrease in T_1_ (P < 0.05) at 0.2 mM, both relative to unlabelled cell pellet.

The difference among T_1_ times of the three CAs is statistically significant at the examined concentrations (0.05–0.2 mM): MnTPPS_3_NH_2_ resulted in a 10.6% to 13.1% (P < 0.05) reduction in T_1_ compared to MnTPPS_4_, which enabled a further 6.1% to 11.1% (P < 0.05) decrease in T_1_ compared to Gd-DTPA. At 0.05 mM, Gd-DTPA-induced decrease in T_1_ from unlabelled control is statistically insignificant (95% confidence interval = [-8.8, 50.6] ms).

**Fig 4:**
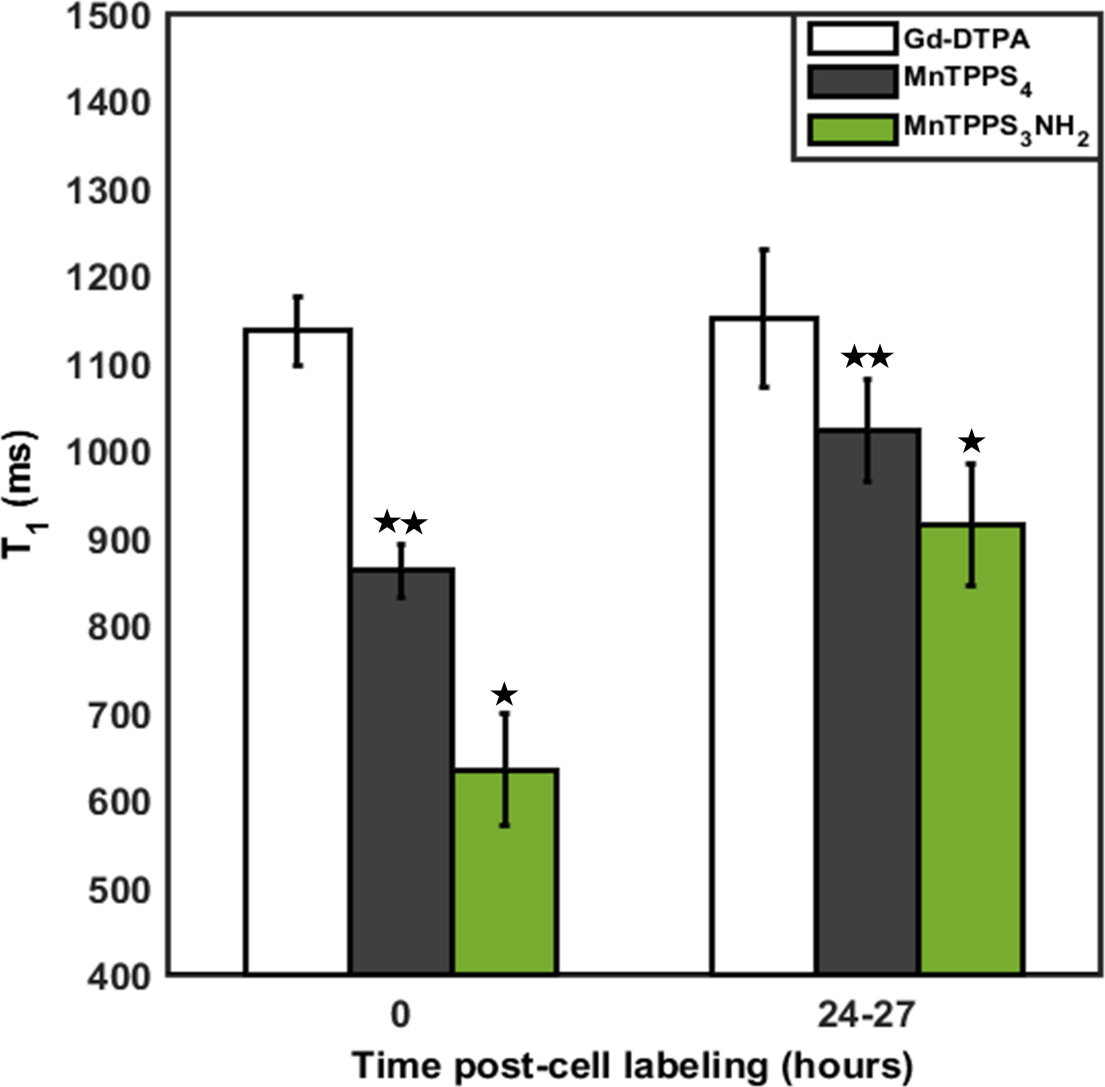
Comparison of CA direct exposure uptake and retention. Averaged T_1_ relaxation times of CA-labelled MDA-MB-231 cell pellets at 0 and 24–27 hrs post-cell labelling for the three CAs at 0.2 mM. Shown are mean values ± standard deviation from pixels within the respective ROIs in Figs 2A and 3A. *Indicates significant difference (P < 0.05) from MnTPPS_4_.** Indicates significant difference (P < 0.05) from Gd-DTPA.

Graph of T_1_ relaxation times of CA-labelled MDA-MB-231 cell pellets at 0 and 24–27 hrs post-cell labelling is shown in Fig 4 for the three CAs at the highest incubation concentration, 0.2 mM. The values represent mean ± standard deviation from all the pixels within each one of the 6 respective ROIs in the quantitative maps of T_1_ relaxation time in Figs 2A and 3A. Compared to Gd-DTPA, MnTPPS_3_NH_2_ enabled more than 44% and 20% (P < 0.05) reduction in T_1_ immediately and 24–27 hrs post-cell labelling, respectively, whereas MnTPPS_4_ resulted in more than 24% and 11% (P < 0.05) decrease in T_1_ immediately and 24–27 hrs post-cell labelling, respectively, both at 0.2 mM.

### Cytotoxicity assessment of CA-labelled breast cell lines

The *in vitro* cytotoxicity assessment results from CA-labelled breast cell lines at 0.2 mM are summarized in Table 3. The values represent the averaged cellular viability, normalized relative to unlabelled cells, from 5 independent measurements ± standard deviation.

**Table 3:** Cytotoxicity results of CA-labelled breast cell lines at 0.2 mM.

Cell line / CA	Gd-DTPA	MnTPPS_4_	MnTPPS_3_NH_2_
MCF-10A	101.6 ± 5.5%	100.7 ± 3.1%	99.7 ± 4.7%
MCF-7	99.2 ± 6.8%	106.7 ± 10.9%	103.6 ± 5.1%
ZR-75-1	100.9 ± 4.6%	102.6 ± 1.0%	102.4 ± 3.3%
MDA-MB-231	99.5 ± 3.0%	104.1 ± 7.3%	99.4 ± 3.2%

CA: contrast agent. The values represent the mean normalized viability, relative to unlabelled cells, from 5 independent measurements ± standard deviation.

### Quantitative MRI for sensitive detection of multiple clinical subtypes of breast cancer

**Fig 5:**
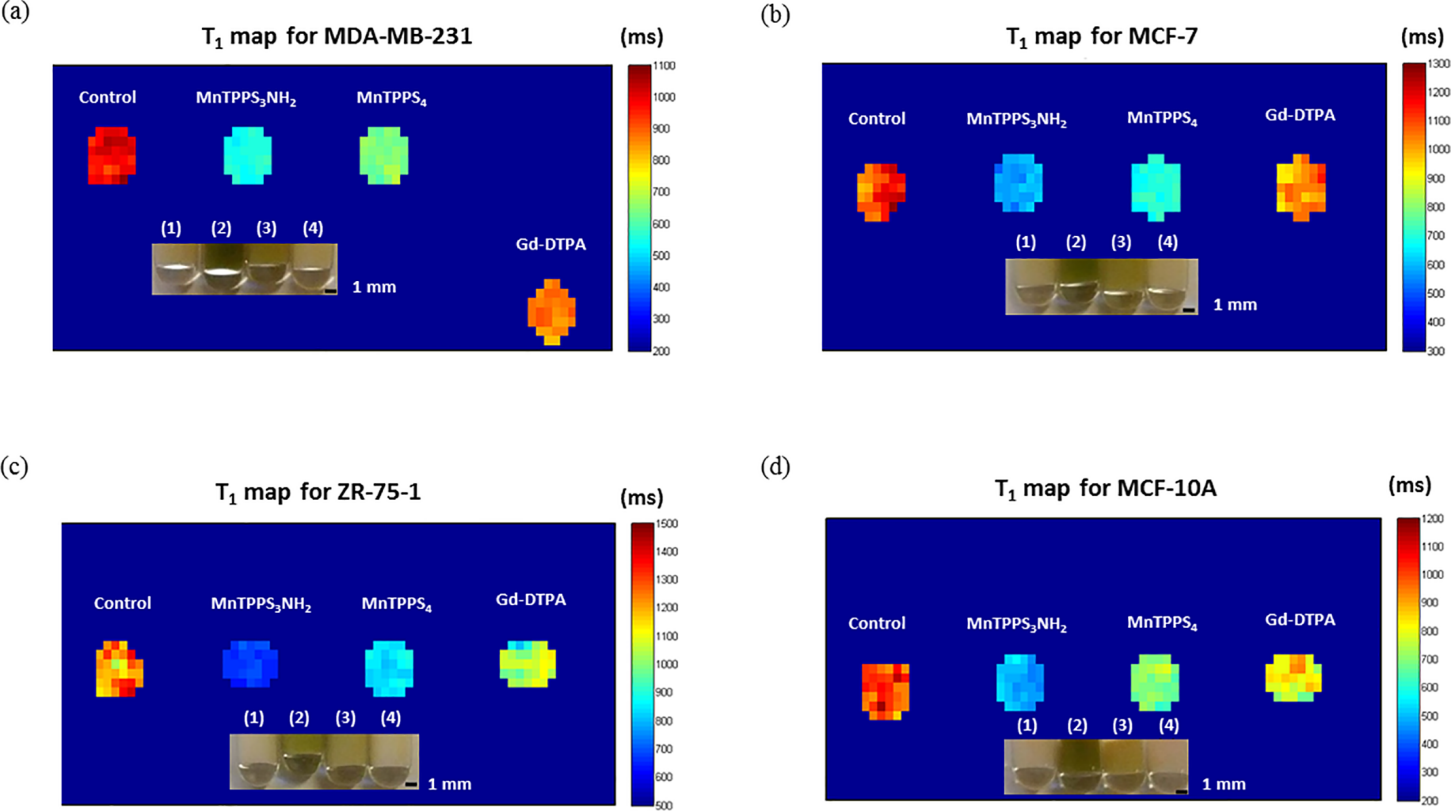
Quantitative maps of T_1_ relaxation times of breast cell lines. (a) MDA-MB-231 (b) MCF-7 (c) ZR-75-1 and (d) MCF-10A cell pellets labelled with MnTPPS_3_NH_2_, MnTPPS_4_, and Gd-DTPA at 0.2 mM. Shown also are pictures of the CA-labelled cell pellets: **(1)** Control; **(2)** MnTPPS_3_NH_2_; **(3)** MnTPPS_4_; **(4)** Gd-DTPA.

Quantitative maps of T_1_ relaxation times for MDA-MB-231, MCF-7, ZR-75-1, and MCF-10A cells are shown in Fig 5A–5D, along with pictures of cell pellets. Table 4 summarizes the T_1_ times of the four breast cell lines. The values represent mean ± standard deviation from at least 30 measurements (pixels) within each ROI in the quantitative maps of T_1_, Fig 5A–5D. Student’s t-test revealed that differences among the cell pellets’ T_1_ times are statistically significant at 95% confidence level. More specifically, relative to Gd-DTPA, MnTPPS_3_NH_2_ enabled highly-sensitive detection of the four breast cell lines by depressing T_1_ by 36.6% (MDA-MB-231), 43.1% (MCF-7), 35.6% (ZR-75-1), and 39.7% (MCF-10A), whereas MnTPPS_4_ decreased T_1_ by 26.0% (MDA-MB-231), 31.7% (MCF-7), 18.5% (ZR-75-1), and 14.3% (MCF-10A).

**Table 4:** T_1_ relaxation times (ms) for CA-labelled cells at 0.2 mM.

Cell line / CA	Control	MnTPPS_3_NH_2_	MnTPPS_4_	Gd-DTPA
MDA-MB-231	992.8 ± 34.5	551.7 ± 15.5*	643.5 ± 20.5**	869.9 ± 26.1
MCF-7	1119.5 ± 77.6	583.8 ± 25.8*	700.0 ± 22.6**	1025.4 ± 54.0
ZR-75-1	1226.4 ± 84.7	667.7 ± 18.4*	845.2 ± 23.7**	1036.7 ± 70.7
MCF-10A	998.1 ± 61.4	489.5 ± 30.6*	695.9 ± 35.1**	812.2 ± 63.6

The values represent mean from at least 30 measurements ± standard deviation.

*Indicates significant difference (P < 0.05) from MnTPPS_4_.

** Indicates significant difference (P < 0.05) from Gd-DTPA.

## Discussion

Because a primary breast tumor mass can be associated with multiple subtypes, possessing diverse biologic profiles, histopathologies, and clinical outcomes [26], it is critical to enable quantitative imaging of various subtypes of breast cancer cells with sufficient sensitivities. Consequently, this work aimed at exploring, and potentially furthering, the oncological capabilities of MRI as a quantitative cellular modality by investigating the potential of MnP-enhanced MRI in detecting multiple clinical subtypes of breast cancer *in vitro*. This promising cellular imaging approach may overcome many limitations associated with conventional Gd-DTPA-enhanced MRI, including narrow post-contrast imaging window.

To compare the degree of positive contrast the examined T_1_ CAs (MnTPPS_3_NH_2_, MnTPPS_4_, and Gd-DTPA) can provide at 3.0T and to determine whether or not they suffer from any T_2_-related negative signal effects, longitudinal and transverse relaxivities, r_1_ and r_2_, were calculated. MnTPPS_3_NH_2_ and MnTPPS_4_ produced an over two-and-a-half-fold and two-fold increase in r_1_, respectively, over Gd-DTPA. Similar fold change was previously reported for MnTPPS_4_ and Gd-DTPA [12,15] and may be attributed, in part, to the anisotropy of the Mn^3+^ ground-state wavefunction in the porphyrin molecule which can bring the spin density of the manganese ions closer to the ^1^H nuclei of the coordinated H_2_O molecules [27]. However, the r_2_/r_1_ ratios for the three CAs are close to unity at relatively low CA concentrations, indicating that the effect of the CAs on T_1_ relaxation times is considerably greater than that on T_2_ due to the inherent longer T_1_ times of biologic tissues.

For the subsequent cell labelling studies, the reductions in T_1_ relaxation times were taken as a direct measure of CA cellular uptake and the resulting MR signal enhancement. This assumption, which has also been applied by others [12,28], is likely to be valid because the relaxation rates in aqueous solution (Fig 1), in aggressive breast cancer cells (Fig 2B), and in tissue extracts [12,29] are proportional to CA concentrations. Moreover, a qualitative observation of the MnP-labelled cell pellets (S4 Fig) reveals a difference in the colour intensity of the pellets which is an evidence that higher cellular uptake of MnP (i.e. darker cell pellet color) translates to a greater T_1_ reduction on MRI. Nevertheless, it should be noted that the relaxivities of CAs can generally be altered by binding to macromolecules [12], distribution of H_2_O protons [29], entrapment in subcellular compartments, and/or simply demetallization. Because the goal was to examine the MR signal enhancement characteristics of the T_1_ CAs, however, the relative changes in the longitudinal relaxation times are the relevant measurements used in this investigation.

The MRI contrast enhancement patterns of the three CAs at 3.0T were investigated by labelling aggressive breast cancer cells, MDA-MB-231, with the CAs at various concentrations and incubation conditions (i.e. direct exposure and cellular retention). The statistically significant differences in T_1_ times across the CA-labelled cell pellets attest to the dose-dependent T_1_ response of the three CAs and suggest that, as mentioned earlier, the longitudinal relaxation rates of the cells are proportional to concentration of CAs and presumably cellular uptake. On the other hand, being T_1_ CAs, their induced dose-dependent changes on T_2_ times are primarily insignificant.

In both the direct exposure and cellular retention experiments, labelling cells with MnTPPS_3_NH_2_ at 0.2 mM yielded the most sensitive detection of MDA-MB-231 cells on T_1_-weighted MRI as it enabled a 53.3% and 26.2% decrease in T_1_ from baseline, respectively. MnTPPS_4_ also offered a sensitive detection by providing a 36.6% (direct exposure) and 17.4% (cellular retention) depression in T_1_ from baseline, whereas Gd-DTPA only provided a 16.5% (direct exposure) and 7.1% (cellular retention) reduction in T_1_ from baseline at 0.2 mM. Despite the slight cellular uptake and positive T_1_ enhancement induced by Gd-DTPA, which has also been reported in previous cell labelling studies [20,21], it is evident from these fundamental investigations that the two MnPs have greater cellular uptake and retention capabilities. The mechanism of this uptake is unknown, although previous work with MnTPPS_4_ suggested that this is not due to interaction with heme-binding protein [28]. However, the observed increase in T_1_ times of all CAs 24–27 hrs post-cell labelling, which corresponds to a decrease in positive signal enhancement on T_1_-weighted MRI, indicates that MDA-MB-231 cells did not fully retain the CAs when incubated in CA-free media. Such an increase in longitudinal relaxation times has also been observed with MnCl_2_-labelled MDA-MB-231 cells incubated in CA-free media in a previous study [6] and may be attributed, in part, to MRI signal dilutions owing to the aggressive cancer cell proliferation.

Subsequent experiments thoroughly assessed the cytotoxicity of the administered CAs and demonstrated that a 24–27 hr labelling of four breast cell lines (MDA-MB-231, MCF-7, ZR-75-1, and MCF-10A) with the three CAs at 0.2 mM had no cytotoxic effects. Therefore, to assess the differential signal-enhancing capabilities of the CAs, MRI was performed after labelling the aforementioned four breast cell lines with the three CAs for approximately 24 hrs at 0.2 mM (direct exposure) and revealed a consistently significant (P < 0.05) sensitivity for cellular detection using MnPs over Gd-DTPA. This is likely due to MnPs’ higher cellular uptake and r_1_ relaxivities at 3.0T. As expected from their chemical structures (S1 Fig), MnTPPS_3_NH_2_ and MnTPPS_4_ exhibited different T_1_ signal enhancement patterns. MnTPPS_3_NH_2_ offered significantly greater (P < 0.05) reductions in T_1_ compared to MnTPPS_4_ in the four breast cell lines possibly due to its amino (-NH_2_) group which makes the agent more hydrophobic, thereby increasing its cellular uptake. This advantage is expected to enable MnTPPS_3_NH_2_ to achieve a positive contrast enhancement similar to MnTPPS_4_ but at lower doses, which can reduce potential systemic toxicity *in vivo*. Moreover, because structural modification of MnTPPS_4_ only occurs at the periphery of the phenyl rings (by replacing one SO_4_ with NH_2_), it causes almost no change on the inner metal binding site of porphyrin and should have little influence on the metal binding stability of MnTPPS_3_NH_2_.

While this work has demonstrated that the two MnPs can enable a substantial positive contrast enhancement of multiple subtypes of breast cancer cells compared to Gd-DTPA, it also revealed that the non-tumorigenic cell line, MCF-10A, exhibited a considerable CA uptake and T_1_ enhancement. The mechanism behind this high CA uptake in MCF-10A cells is unclear and requires future investigation of the subcellular localization of porphyrins in tumorigenic and non-tumorigenic cell lines. Moreover, to confirm that the high positive signal enhancement obtained indeed corresponds to cellular uptake of paramagnetic CAs, it is suggested that future cellular MRI studies will incorporate an analytical method of absolute intracellular element quantification (e.g. inductively coupled plasma mass spectrometry, ICP-MS). However, it should be noted that the co-authors have evidence from ICP in many of the previous MnP studies that higher MR signal correlates positively with Mn ion content on a per-cell basis. Because this observation has been very repeatable in our studies [6,30,31], we can confidently use the MR signal as an imaging correlate of ion content.

While future *in vivo* work would be helpful to elucidate the pharmacokinetics and tumor contrast enhancement efficiency of the contrast agents, the goal of the current work was to demonstrate the capability MnPs in detecting multiple clinical subtypes of breast cancer cells. *In vitro* experiments on cell lines provide a more controlled setting to examine cell line-specific uptake of contrast agents. *In vivo* validation in animal models is an important next step but would be inappropriate as a first platform for testing because the *in vivo* environment presents many other confounders, such as differences in take-rates, that can obscure the scientific question we are pursuing at hand.

It is important to note that often times when new compounds are being discovered, there can be a bias for one compound over another because one property of the compound provides a greater advantage. In the case of Gd versus Mn, the Gd has a higher efficiency because it has 7 unpaired electrons compared to Mn, which has 5 unpaired electrons. For this reason alone, Gd is the champion, despite the fact that it is toxic. Furthermore, it is easier to chelate Gd more stably than Mn, which is a neurotoxin in its ionic form. However, with the Mn-based contrast agents presented in this paper, the porphyrin ring provides a very high thermodynamic stability. With the recent recognition that Gd is toxic and has been associated with safety concerns in patients, there is a renewed interest in finding alternatives to Gd. MnPs present a viable alternative.

## Conclusions

Current oncological applications of Gd-DTPA-enhanced MRI rely on bulk manifestations in tumor morphology, imaged within a relatively short window, rather than early, pre-angiogenic changes at the cellular level driving these manifestations. Using quantitative cellular MRI, this work has demonstrated the potential of MnPs for sensitive imaging of multiple clinical subtypes of breast cancer cells due to their high relaxivities and cellular uptake and retention compared to Gd-DTPA, despite the observed positive enhancement from a non-tumorigenic cell line. This study has also demonstrated, for the first time, the potential of the more hydrophobic MnP, MnTPPS_3_NH_2_, as a T_1_ CA for sensitive detection of various breast cancer cells *in vitro* using quantitative cellular MRI.

## Supporting information

S1 Fig The chemical structures of (a) MnTPPS_4_ and (b) MnTPPS_3_NH_2_.
(TIF)

S2 Fig Schematic diagram for the arrangement of replicates in the cytotoxicity assessment of CA-labelled breast cell lines in a 96-well plate. (TIF)

S3 Fig The ultraviolet-visible spectra for (a) MnTPPS_4_ and (b) MnTPPS_3_NH_2_.
(TIF)

S4 Fig Picture of MnTPP_3_NH_2_-labelled MDA-MB-231 cell pellets at (1) 0.2 mM, (2) 0.1 mM, and (3) 0.05 mM.
(TIF)
