# Correction: Manganese-porphyrin-enhanced MRI for the detection of cancer cells: A quantitative *in vitro* investigation with multiple clinical subtypes of breast cancer

**DOI:** 10.1371/journal.pone.0206720

**Published:** 2018-11-12

**Authors:** The PLOS ONE Staff

The name of the sixth author appears incorrectly in the citation. The correct citation is: Alhamami M, Cheng W, Lyu Y, Allen C, Zhang X-a, Cheng HL (2018) Manganese-porphyrin-enhanced MRI for the detection of cancer cells: A quantitative *in vitro* investigation with multiple clinical subtypes of breast cancer. PLoS ONE 13(5): e0196998. https://doi.org/10.1371/journal.pone.0196998
