# Supplementary figures and images for: Manganese-porphyrin-enhanced MRI for the detection of cancer cells: A quantitative in vitro investigation with multiple clinical subtypes of breast cancer
Source: PLoS One. 2018 May 24;13(5):e0196998. doi: 10.1371/journal.pone.0196998 (PMC5993062; doi:10.1371/journal.pone.0196998)

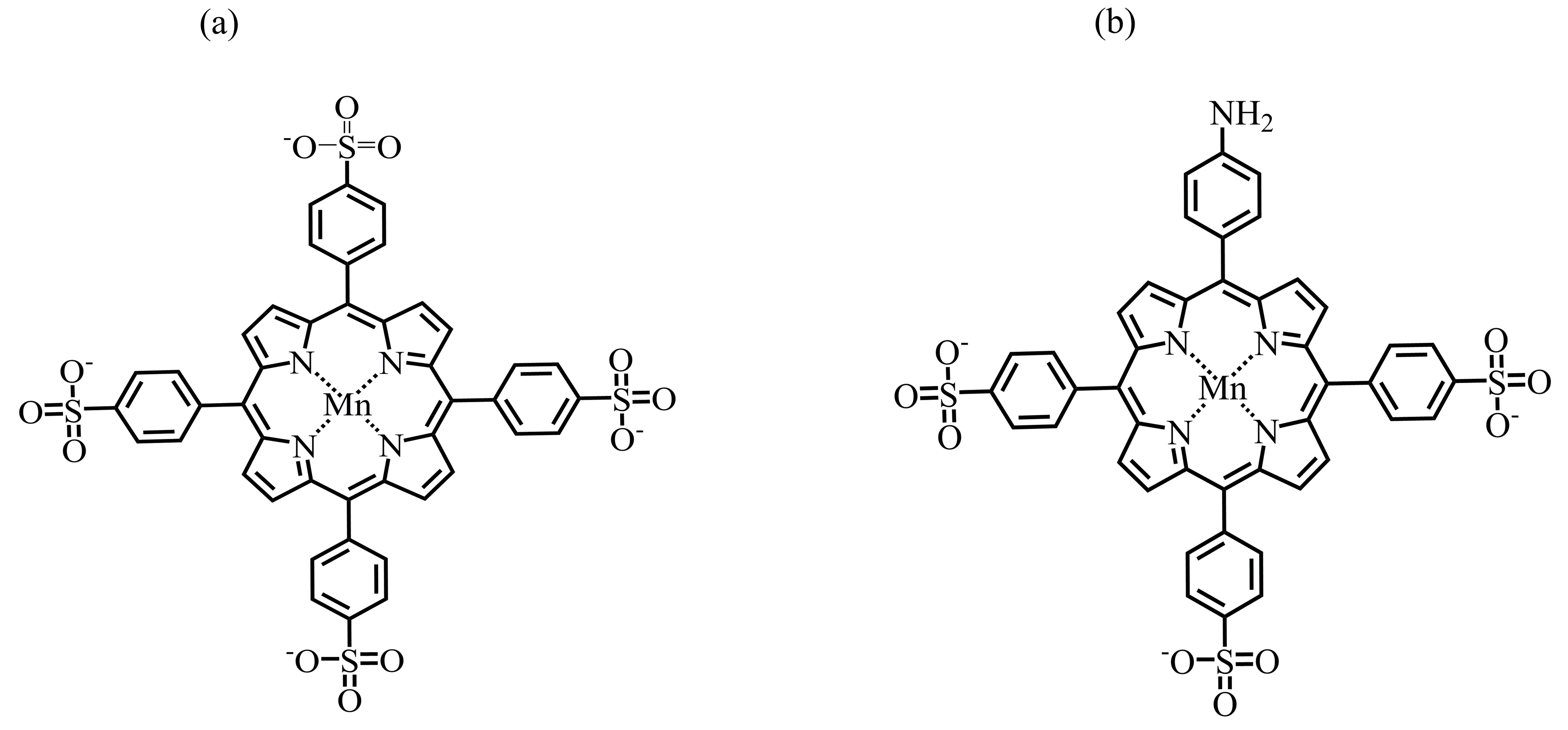

Supplement: S1 Fig — The chemical structures of (a) MnTPPS4 and (b) MnTPPS3NH2. (TIF) [file pone.0196998.s001.tif]

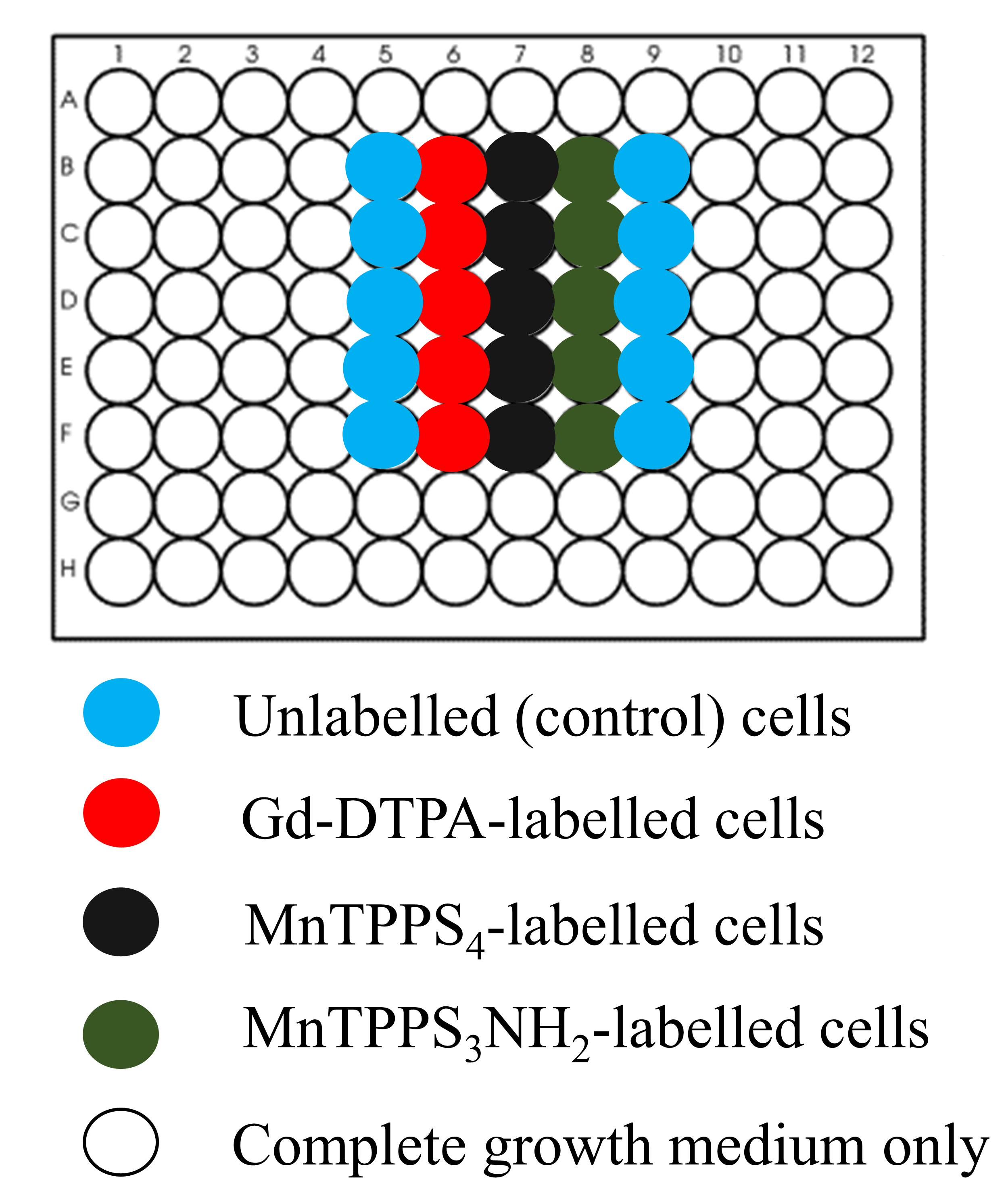

Supplement: S2 Fig — (TIF) [file pone.0196998.s002.tif]

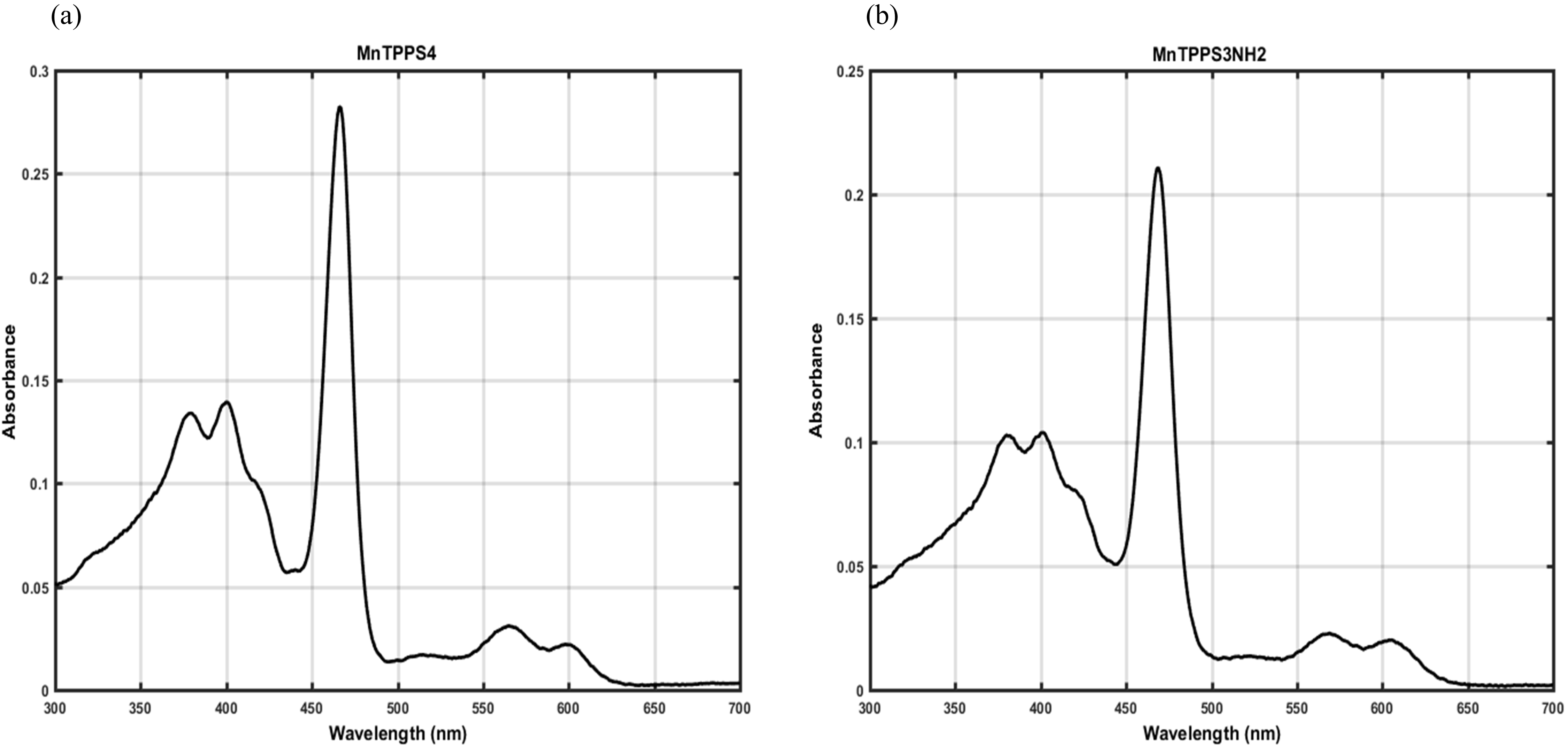

Supplement: S3 Fig — The ultraviolet-visible spectra for (a) MnTPPS4 and (b) MnTPPS3NH2. (TIF) [file pone.0196998.s003.tif]

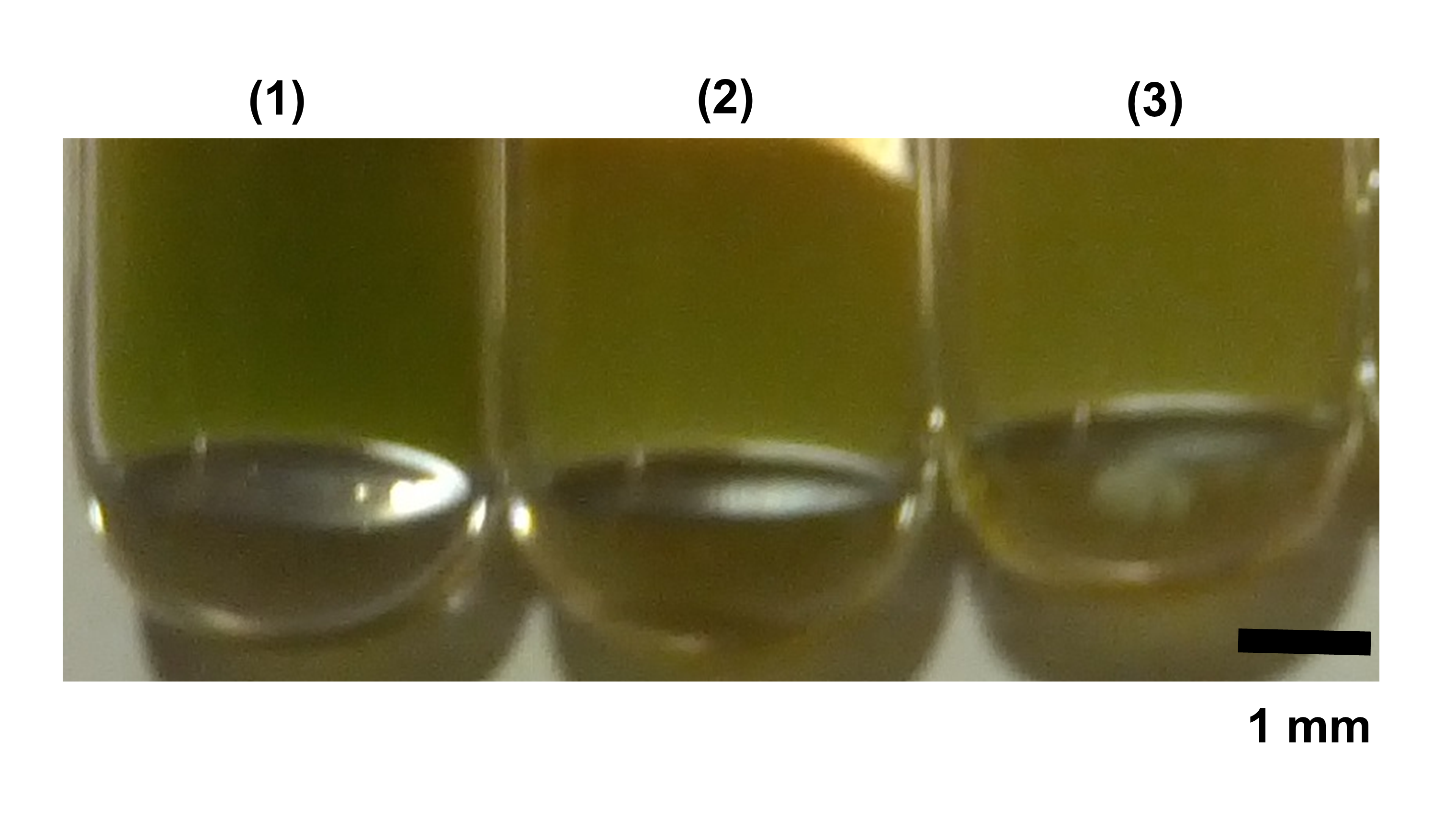

Supplement: S4 Fig — Picture of MnTPP3NH2-labelled MDA-MB-231 cell pellets at (1) 0.2 mM, (2) 0.1 mM, and (3) 0.05 mM. (TIF) [file pone.0196998.s004.tif]
